# The Role of QRS Complex and ST-Segment in Major Adverse Cardiovascular Events Prediction in Patients with ST Elevated Myocardial Infarction: A 6-Year Follow-Up Study

**DOI:** 10.3390/diagnostics14101042

**Published:** 2024-05-17

**Authors:** Srđan Maletin, Milovan Petrović, Anastazija Stojšić-Milosavljević, Tatjana Miljković, Aleksandra Milovančev, Ivan Petrović, Isidora Milosavljević, Ana Balenović, Milenko Čanković

**Affiliations:** 1 Faculty of Medicine, University of Novi Sad, 21000 Novi Sad, Serbia; srdjan.maletin@mf.uns.ac.rs (S.M.); milovan.petrovic@mf.uns.ac.rs (M.P.); anastazija.stojsic@mf.uns.ac.rs (A.S.-M.); tatjana.miljkovic@mf.uns.ac.rs (T.M.); aleksandra.milovancev@mf.uns.ac.rs (A.M.); 014662@mf.uns.ac.rs (I.P.); 014568@mf.uns.ac.rs (I.M.); 014631@mf.uns.ac.rs (A.B.); 2 Institute for Cardiovascular Diseases of Vojvodina, 21204 Sremska Kamenica, Serbia

**Keywords:** ST-elevation myocardial infarction, acute coronary syndrome, QRS complex, ST-segment, ECG, percutaneous coronary intervention

## Abstract

Background: as a relatively high number of ST-segment elevation myocardial infarction (STEMI) patients develop major adverse cardiovascular events (MACE) following percutaneous coronary intervention (PCI), our aim was to determine the significance, and possible predictive value of QRS complex width and ST-segment elevation. Methods: our patient sample included 200 PCI-treated STEMI patients, which were divided into two groups based on the following duration of symptoms: (I) less than 6 h, and (II) 6 to 12 h. For every patient, an ECG was performed at six different time points, patients were followed for up to six years for the occurrence of MACE. Results: the mean age was 60.6 ± 11.39 years, and 142 (71%) were male. The 6–12 h group had significantly wider QRS complex, higher ST-segment elevation, lower prevalence of ST-segment resolution as well as MACE prevalence (*p* < 0.05). ECG parameters, QRS width, and magnitude of ST-segment elevation were proved to be independent significant predictors of MACE in all measured time points (*p* < 0.05). Even after controlling for biomarkers of myocardial injury, these ECG parameters remained statistically significant predictors of MACE (*p* < 0.05). Conclusion: our study highlights that wider QRS complex and a more pronounced ST-segment elevation are associated with longer total ischemic time and higher risk of long-term MACE.

## 1. Introduction

Even though there have been improvements in the management of ST-segment elevated myocardial infarction (STEMI), as the most serious subtype of coronary artery disease, it still represents a significant factor in global mortality [1]. Percutaneous coronary intervention (PCI) and pharmacological treatments have improved survival rates for patients with STEMI (ST-elevation myocardial infarction). However, in patients who survive the acute phase of STEMI, major adverse cardiovascular events (MACE) [2,3] represent a substantial cause of morbidity and mortality, with a reported prevalence of to 15%, even after a successful PCI procedure. Current recommendations emphasize the need for effective measures in MACE risk reduction [3,4]. The incidence of MACE ranges from 4.2% to 51%, and some of the risk factors are established atherosclerotic disease, triple vessel disease, stent implantation, hypertension, uric acid levels, and estimated glomerular filtration rate (eGFR) [4,5,6]. In patients undergoing PCI, short-term mortality has been extensively studied. However, long-term mortality remains an understudied research objective [7].

The 12-lead electrocardiogram (ECG) represents the most utilized method for diagnosing, triaging, and determining appropriate treatment in STEMI patients [8]. As one of the most commonly used methods in clinical practice, it allows for an immediate risk assessment of acute coronary syndrome patients [9,10]. Even though it is a well-established diagnostic tool, different ECG presentations of acute myocardial ischemia, in terms of prognosis, are not well defined [9]. The QRS complex morphology, and ST-segment changes, have already been described as potential predictors of certain outcomes. The authors analyzed the role of poor R wave progression, with a dominant QS pattern in V3, in reduced left ventricular ejection fraction (LVEF) prediction, as well as the impact of QRS complex width on left ventricular systolic dysfunction (LVSD), heart failure (HF), and death [11,12]. Lately, fragmented QRS (fQRS) and QRS distortion, emerged as novel predictors of reperfusion success [8].

Based on ST-segments resolution, we can identify successful recanalization of infarct-related arteries (IRA) after thrombolytic therapy in acute myocardial infarction patients [13], as well as in those patients with reduced chance of receiving benefits from early flow restoration in IRA [14].

Although ECG is commonly used in clinical practice, its elements such as the QRS complex and ST-segment have not been extensively evaluated as MACE predictors in STEMI patients. Therefore, the aim of this study was to investigate the potential of these ECG elements as predictors of long-term MACE in STEMI patients.

## 2. Materials and Methods

### 2.1. Sample and Group Formation

This longitudinal prospective cohort study was conducted at the tertiary cardiovascular center and was approved by the ethics committee of the institution. The study included patients that were hospitalized with a principal diagnosis of STEMI from 1 January 2016 to 31 December 2018.

The inclusion criteria for this study were the following: (I) patients older than 18 years, (II) patients with first presentation of acute myocardial infarction with ST-segment elevation treated with PCI, and (III) the symptoms duration lasted less than 12 h.

The exclusion criteria were the following: (I) additional comorbidities, which could influence the patients’ outcomes, (II) patients with previous myocardial infarction, with or without ST-segment elevation, (III) structural heart diseases, (IV) patients with a previously performed surgical revascularization procedure, (V) patients in the state of cardiogenic shock during the admission, (VI) left or right bundle branch block detected during the admission, (VII) patients with the atrioventricular block of the second or third degree, (VIII) patients with a permanent or temporary pacemaker, (IX) patients with disbalance of electrolytes that could influence the ECG (e.g., calcium and potassium levels), (X) contraindications for the administration of antiplatelet drugs, (XI) patients with no need for further intervention after coronary angiography was performed, and (XII) patients who were not compliant. The data regarding the patient’s risk factors, comorbidities, clinical findings at admission, length of hospitalization, echocardiographic findings, and intrahospital mortality were collected from the electronic medical records. follow-up data were collected during the regular check-up examinations of the patients.

Consecutive patents who fulfilled inclusion and exclusion criteria were included in the study. Based on the duration of the symptoms to balloon time, patients were divided into the following two groups: (I) Group A, in which symptoms lasted up to 6 h from the onset to the balloon, and (II) Group B, in which symptoms lasted from 6 to 12 h from the onset to the balloon. The target number of the patients was same for the both groups and it was one hundred.

On admission and during index hospitalization the following data were collected: (I) demographic data, (II) functional status, assessed through the New York Heart Association (NYHA) classification, Killip classification, and Canadian Cardiovascular Society (CCS) angina scale, (III) data about myocardial infarction localization, culprit vessel, and performed treatment, (IV) laboratory data, measuring creatine kinase isoenzyme MB (CK-MB) and troponin I (TnI), (V) hospitalization data, (VI) echocardiographic findings, and (VII) ECG data.

### 2.2. Electrocardiographic Data

Electrocardiography (ECG) was performed for every patient. Recording was performed on a Schiller AT-102, Baar, Switzerland ECG machine at the speed of 50 mm/s and a gain of 10 mm/mV to obtain a high precision rate. The ECG parameters were measured manually using a graduated lens. The sum of the ST elevations was measured in all 12 derivations in three consecutive cardiac cycles and then averaged. The measurements were taken at the following different time points: (I) on admission, prior to the PCI procedure, (II) immediately after the PCI procedure, (III) an hour after the PCI procedure, (IV) on the third day of hospitalization, (V) at the one month follow-up, and (VI) at the six months follow-up. The ST-segment resolution was defined as a decrease of more than 50% of the initial ST-segment elevation, measured in millimeters.

### 2.3. Coronary Angiography

All patients underwent urgent coronary angiography which was performed at the Siemens Artis zee Forchheim, Germany, catheterization laboratory. Usually, the right radial artery was the preferred access point for vascular entry. However, if this was not possible, the femoral artery was used. The coronary ostia were then cannulated, and contrast was injected to assess the coronary arteries. Significant stenoses were major epicardial vessels or their branches with stenosis greater than 70%, or left main stenosis greater than 50%, which required intervention. These vessels underwent PCI with percutaneous transluminal coronary angioplasty (PTCA) with or without stenting. The stents used were drug-eluting stents.

### 2.4. Echocardiography

All subjects underwent two-dimensional transthoracic echocardiography (ECHO) using a Vivid E9 (GE Healthcare, Milwaukee, WI, USA) machine that was equipped with an M5S-D, 1.5–4.6 MHz transducer with continuous ECG monitoring during the examination. All measurements and calculations were performed via the previously described methods according to the standardized procedures of the European Association of Cardiovascular Imaging and the American Society for Echocardiography. Different parameters were measured and compared [15].

### 2.5. Patient Follow-Up

The total follow-up period was six years with a mean follow-up period of 2025.5 (1964–2395.5) days, during which data were collected at two-time points of six months (short-term) and at the end of the study after six years of the study onset (long-term). For the short-term follow-up, patients were assessed during the regular exams, while for the long-term outcome follow-up, patients were contacted via telephone. During this time, the absolute number of losses in follow-up patients was 23, including 14 in group A, and 9 in group B, without a statistically significant difference (*p* = 0.380). After discharge, all patients were followed up for the occurrence of major adverse cardiac events (MACE), which included the following: myocardial reinfarction, the need for an additional PCI procedure of both culprit and non-culprit vessels, heart failure, stroke, and cardiovascular death. For this part of the analysis, patients were divided into the following two groups: (I) patients with MACE, and (II) patients without MACE.

### 2.6. Statistical Analysis

The collected data were divided into subcategories, based on their similarities, and were analyzed on the following three levels: (I) the whole sample, (II) patients with symptoms duration of more than 6 h (Group A), and (III) patients with symptom duration of less than 6 h. Python 3.10.6 was used as the programming language in this research. Categorization of the variables followed, in which the division was made into categorical and continuous groups, and variables were analyzed using adequate tests. Categorical variables were expressed as numbers (percentages), and the Chi-squared test was used to determine differences between the two groups. Continuous variables were presented as medians (interquartile range) or mean ± standard deviation (SD) and analyzed via Student t-test or Mann–Whitney U test, based on sample normality using Kolmogorov–Smirnov test. Two-tailed tests were used, and statistical significance was observed at a level *p* < 0.05, for every variable. Using binary regression analysis, the odds ratios of developing adverse cardiovascular events were determined. The predictive value of ECG measurements was determined via the area under the receiver operator characteristic (AUC-ROC) curve.

## 3. Results

### 3.1. Baseline Patients Characteristics

In Table 1, data from index hospitalization are presented. Out of 200 patients with a mean age of 60.6 ± 11.39 years, 142 were male and 58 were female. STEMI patients had high prevalence of hypertension 63%, 32% had family history of cardiovascular diseases, and 18% had diabetes. The group with symptoms duration of less than 6 h had a significantly lower heart rate 77.5 bpm (IQR 65–85) vs. 79 bpm (IQR 70.0–86.25), *p* = 0.035). The most prevalent infarct-related artery (IRA) differed between groups since the right coronary artery was more common in group A, while the left anterior descending artery was more common in group B. Markers of myocardial injury (CK-MB and TnI) were significantly higher in group B, during all four measurements. Length of hospitalization was not significantly different between the observed groups (six days (IQR 5–7) vs. five days (IQR 5–7), *p* = 0.265). The left ventricle ejection fraction value was significantly lower in group B, (50.0% (IQR 46.0–55.0) vs. 48.0% (IQR 42.0–55.0), *p* = 0.024), and 6-month follow-up (55.0% (IQR 50.0–57.5) vs. 50.0% (IQR 45.0–55.0), *p* = 0.001). Additional information about the PCI procedure and culprit vessels are stored in Table S1.

After the inclusion, 3% of patients had deteriorated.

### 3.2. ECG Data

#### 3.2.1. QRS Complex Width Measurement

The QRS complex width were significantly shorter in group with symptoms duration of less than 6 h (Table 2), 60 min post-PCI (86.5 msec (IQR 80.0–100.0) vs. 100.0 msec (IQR 87.25–100.0), *p* = 0.008), 72-h post-PCI (81.0 msec (IQR 80.0–90.5) vs. 95.0 msec (IQR 85.0–100.0), *p* = 0.001), one month after (80.0 msec (IQR 80.0–90.0) vs. 90.0 msec (IQR 85.0–100.0), *p* = 0.001), and six months after (80.0 msec (IQR 80.0–90.0) vs. 90.0 msec (IQR 80.0–100.0), *p* = 0.001).

#### 3.2.2. ST-Segment Elevation

ST-segment elevation was lower in group with symptoms’ duration of less than 6 h (Table 2) 60 min post-PCI (0.5 mm (IQR 0.0–1.0) vs. 1.0 mm (IQR 0.0–2.0), *p* = 0.005), 72-h post-PCI (0.0 mm (IQR 0.0–0.5) vs. 0.5 mm (IQR 0.0–1.0), *p* = 0.001), one month after (0.0 mm (IQR 0.0–0.0) vs. 0.0 mm (IQR 0.0–0.5), *p* = 0.003), and six months after (0.0 mm (IQR 0.0–0.0) vs. 0.0 mm (IQR 0.0–0.0), *p* = 0.013). After the PCI procedure, ST-segment resolution was significantly more prevalent in group A compared to group B (72% vs. 49%, *p* = 0.001). However, there were no significant differences observed between the groups at other time points.

### 3.3. Follow-Up Data

#### 3.3.1. 6-Month Follow-Up

Out of 200 patients, 32 had MACE during a 6-month period. MACE was more common in group B (9% vs. 23%, *p* = 0.012). The most frequent was heart failure (*n* = 28), which was more abundant in group B (6% vs. 22%, *p* = 0.002). During this period, six patients died. The majority of them (*n* = 5) died out of the hospital, while one died during the hospitalization. All patients died due to a coronary event (Table 3). Additional data are stored in Table S2.

#### 3.3.2. 6-Year Follow-Up

During this extensive follow-up period, 44 patients had MACE. The most common one was cardiovascular death (*n* = 14), followed by re-PCI of the non-culprit vessel (*n* = 10), and reinfarction (*n* = 7). Taking into consideration the time frame from the PCI procedure until the MACE, it was determined that MACE happened earlier in group B (2287 days (IQR 1874–2476) vs. 1854 days (IQR 615–2208), *p* = 0.001). All follow-up data are presented in Table 3.

### 3.4. ECG Data in MACE Prediction

#### 3.4.1. Value of QRS Complex Width in Association with MACE

**Figure 1:**
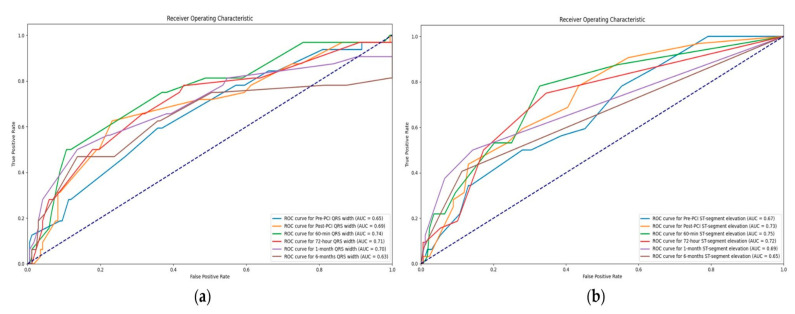
Receiver operator characteristic curves for: (**a**) QRS complex width, based on the time point of measurement; (**b**) ST-segment elevation, based on the time point of measurement.

A wider QRS complex was significantly associated with a higher possibility of developing a 6-year MACE, at all time points of measurement. A QRS width increase of 1 msec above 99 msec width after a month increased the risk of MACE for 4.2% (OR = 1.042, 95% CI 1.012–1.074, *p* = 0.007). An increase in pre-PCI QRS complex width by 1 msec above 99 msec width increased the risk of MACE by 3% (OR = 1.028, 95% CI 1.003–1.053, *p* = 0.029). All results are presented in Table 3. The highest AUC value was displayed by the QRS complex 60 min after the PCI procedure (AUC = 0.74) (Figure 1a).

#### 3.4.2. Value of ST-Segment Elevation in Association with MACE

During all time points of measurement, a higher ST-segment elevation was associated with a higher possibility of developing a 6-year MACE (Table 4), *p* = 0.001, *p* = 0.001, *p* = 0.001. The strongest predictor was the one-month ST-segment elevation (OR = 3.256, 95% CI 1.572–6.747, *p* = 0.001). An increase in ST-segment elevation after one month by 1mm increased the risk of MACE by 3.26 times. All results are presented in Table 5. The highest AUC values were demonstrated 60 min after the PCI (AUC = 0.75) (Figure 1b).

In the model where QRS width pre-PCI controlled for CPK and TNI QRS width remained significant (Table 6).

The same was observed when ST-segment elevation was controlled for CPK and TNI (Table 7). ST elevation pre-PCI increased for 1 mm controlled for troponin and CPK increased the risk of MACE by 16% (OR = 1.16, 95%CI 1.02–1.32, *p* = 0.019).

## 4. Discussion

To the best of our knowledge, this is the first study that analyzed the possible utilization of QRS complex width and ST-segment elevation in long-term MACE prediction. Patients showing symptoms lasting from 6 to 12 h had higher levels of myocardial injury markers (CK-MB and TnI) and their echocardiograms revealed lower left ventricle EF values, indicating reduced myocardial function. This group also exhibited wider QRS complex, higher ST-segment elevation at almost all time points with a lower prevalence of ST-segment resolution and a higher prevalence of MACE. Both the width of the QRS complex and the elevation of the ST-segment were significantly associated with major adverse cardiac events (MACE) at all measured time points. An increase of 1msec of pre-PCI QRS complex width significantly increased the risk of 6-year MACE by 2.8%, and an increase in 1mvol pre-PCI ST-segment elevation increased the risk by 61%.

The group with a shorter onset-to-treatment time (OTT) exhibited lower HR. In previously published research, the increased elevated admission HR in PCI-treated STEMI patients was a strong, independent predictor of adverse outcomes and mortality. A value of HR greater than 80 beats per minute (bpm) could identify patients with an increased risk of death. However, it is not certain whether HR reduction results in improved outcomes [15,16,17]. In the available literature, patients with longer symptom onset-to-door time (SODT) had poorer prognoses and worse survival rates. Some of the factors influencing SODT may be older age, ethnicity, and diabetes mellitus, as well as the technical aspect of healthcare-providing [18]. It was shown that during ‘’on-hours’’ (work days), patients more commonly contacted physicians, rather than the emergency services, which led to increased SODT [19]. As time-to-treatment is directly associated with infarct size, minimizing any component of treatment delay and emphasizing shorter onset-to-treatment time in STEMI patients is imperative for decreasing mortality and improving outcomes [20,21]. In our patient group with longer OTT, left ventricular ejection fraction (LVEF) was significantly lower, possibly due to the more extensive myocardial involvement. Decreased LVEF is an important predictor of cardiac prognosis, and reduced values are seen in up to 40% of STEMI patients [22,23].

In our sample, a significantly wider QRS complex was observed in the group with longer OTT, at almost all time points of measurement. In previous research, changes in the QRS complex and the value of the QRS score were associated with the size of the infarcted area [24,25]. As longer OTT leads to more extensive myocardial necrosis [20], our findings are expected as larger infarction results in wider QRS complex. These findings are in line with the current literature, as QRS complex and its elements are established predictors of cardiac risk, mortality, and outcomes of STEMI patients [8,26,27]. However, QRS width has not been extensively studied as a predictor of adverse events. In our analysis, ST-segment elevation measured 72 h after the PCI procedure showed the highest statistical difference between the observed groups. Similar to the previous parameter, ST-segment elevation was more significantly pronounced in the longer OTT group, while ST-segment resolution was more commonly absent in the same group. Lack of ST-segment resolution, as well as higher elevation, were previously associated with larger infarct size, leading to worse patient outcomes, and lower LVEF [28,29,30,31,32,33]. As total ischemic time (TIT) was longer in the previously mentioned group, this finding strongly supports the idea that the infarct volume highly depends on the ischemic time.

During long-term follow-up, 24.6% of patients had MACE. It was more common in the longer OTT group, and the most prevalent type was cardiovascular death, followed by re-PCI of the non-culprit vessel. There is a wide range of MACE prevalence rate in the reported literature of 4.2% to 51%. Risk factors, such as established atherosclerotic disease, triple vessel disease, stent implantation, hypertension, diabetes, and decreased kidney function, were showed to be significant predictors of MACE [4,5,6]. In our cohort, there was no statistically significant difference observed between the risk factors, so the main possible explanation could be prolonged ischemic time, resulting in more extensive myocardial lesions. Even though reperfusion is the main therapeutic aim in STEMI patients, it is well known that it can be followed by a myocardial reperfusion injury (MRI) [34]. Myocardial damage during an acute myocardial infarction is a result of an interplay between ischemia and following reperfusion. Consequently, numerous conditions arise, such as ventricular arrhythmias, myocardial stunning, microvascular obstructions (MVO), as well as intramyocardial hemorrhage. All of these can significantly influence patient outcomes [35]. The size of the myocardial infarction and MVO represent independent predictors for long-term mortality, as well as the development of HF in these patients [36,37]. According to the latest European Cardiology Society (ESC) guidelines for acute coronary syndrome treatment, the strategies for reducing ischemic-reperfusion lesions are still an unfulfilled clinical need [38], as well as a potential focus of future research. Due to extensive myocardial injury, leading to previously described changes in QRS complex width, and ST-segment elevation, these ECG elements have demonstrated certain predictive power, in terms of MACE. The highest AUC value was shown by the QRS complex (AUC = 0.74) width and ST-segment elevation (AUC = 0.75) 60 min after the PCI procedure.

Deng W. et al. in recent publication concluded that there is the rising number of modeling studies with questionable predictability and that there is a need for external validation. Our paper analyzed two ECG parameters whose utility in practice is easy and simple. Both ECG parameters, QRS width and magnitude of ST-segment elevation, were proved to be independent significant predictors of MACE in all measured time points. Even after controlling for biomarkers of myocardial injury, these ECG parameters remained statistically significant predictors of MACE. We hypothesize that these simple ECG parameters could be used on admission, during hospitalization, and in follow-up to identify patients with increased risk of MACE. When analyzing ROC curves, these parameters showed moderate sensitivity and specificity, however, we found that there was a good correlation of these parameters with MACE. One of the biggest limitations in these parameters is that they can be affected by conduction system disorders, etc. Therefore, these parameters should be used in patients with the first manifestation of STEMI without prior structural and rhythmical disorders. To the best of our knowledge, this is the first article to analyze the dynamics of ST-segment resolution and QRS complex width simultaneously as predictors of long-term adverse events. Previous studies, such as those by van der Zwaan and Ndrepepa, have analyzed early changes in ST-segment resolution after pPCI. However, their findings show no impact on long-term outcomes. It is worth noting that these studies only observed the ECG for a period of 120 min. Nevertheless, future research and external validation might include these easily measured ECG parameters in predictive models, where their real predictive power would be obtained [39,40,41].

## 5. Conclusions

To the best of our understanding, this study represents pioneering research that utilized QRS complex width and ST-segment elevation in the context of predicting long-term major adverse cardiovascular events. Wider QRS complex and more pronounced ST-segment elevation were associated with longer total ischemic time (TIT) at all time points of measurement, accompanied by the absence of ST-segment resolution. Longer ischemic time led to more frequent manifestations of a 6-year MACE, which developed in a shorter time frame compared to the group of patients in which the PCI procedure was completed in less than 6 h. QRS width and magnitude of ST-segment elevation were proved to be independent significant predictors of MACE in all measured time points.

## Figures and Tables

**Table 1:** Basic ST-segment elevation myocardial infarction (STEMI) patients’ characteristics.

Variables	Whole Sample (*n* = 200) *n* (%) Mean ± SD Median [IQR]	Symptoms Duration of Less than 6 h (*n* = 100) Median [IQR] %	Symptoms Duration of Less than 6 h (*n* = 100) Median [IQR] %	*p* Value
Sex				1.000
Female	58 (29%)	29	29	
Male	142 (71%)	71	71	
Age (years)	60.6± 11.39	58.5 [52.0–67.0]	60.0 [52.0–74.0]	0.154
Height (cm)	173.1 [165–180]	175.0 [166.75–180.0]	175.0 [165.0–180.0]	0.463
Weight (kg)	82.6 [75–90]	82.0 [75.0–90.0]	80.0 [70.0–90.0]	0.299
Body mass index (kg/m^2^)	27.5 [24.7–29.4]	26.8 [25.24–29.55]	26.45 [24.35–29.4]	0.426
Body surface area (m^2^)	2.0± 0.2	2.0 [1.87–2.08]	1.98 [1.83–2.12]	0.301
Heart rate (beats/min)	78.5 [65–85]	77.5 [65.0–85.0]	79.0 [70.0–86.25]	0.035
Systolic blood pressure (mmHg)	141.0 [126.3–153.8]	140.0 [120.0–150.0]	140.0 [130.0–160.0]	0.131
Diastolic blood pressure (mmHg)	82.4 [70–90]	80.0 [70.0–90.0]	80.0 [80.0–90.0]	0.437
Vascular risk factors and comorbidities				
Hypertension	126 (63%)	65	61	0.660
Diabetes mellitus	36 (18%)	17	19	0.854
Family history	63 (32%)	32	31	1.000
Kidney function				
Creatinine (micromol/L)	96.6 ± 24.1	93.0 [79.0–108.25]	94.0 [81.75–106.25]	0.829
Clearance (mL/min)				
>90	89 (45%)	50	39	0.155
60–90	80 (40%)	40	40	1.000
45–59	23 (12%)	7	16	0.076
30–44	4 (2%)	2	2	1.000
15–29	4 (2%)	1	3	0.614
Duration of the symptoms (min)	304.3 [120–420]	120.0 [90.0–180.0]	420.0 [360.0–600.0]	0.001
Killip				
1	128 (64%)	72	56	0.027
1/2	4 (2%)	1	3	0.614
2	56 (28%)	24	32	0.270
2/3	6 (3%)	1	5	0.214
3	4 (2%)	0	4	0.130
4	2 (1%)	2	0	0.477
Post-MI NYHA status				
1	175 (88%)	89	86	0.669
2	18 (9%)	11	7	0.459
2/3	2 (1%)	0	2	0.477
3	1 (0.5%)	0	1	1.000
4	4 (2%)	0	4	0.130
Post-MI CCS				
0	188 (94%)	95	93	0.766
1	9 (5%)	4	5	1.000
2	1 (1%)	0	1	1.000
3	1 (1%)	0	1	1.000
4	1 (1%)	1	0	1.000
Myocardial injury assessment				
Creatine Kinase isoenzyme MB (CKMB)				
Pre-PCI	61.5 [24–68.5]	27.5 [21.0–35.25]	59.0 [36.0–110.0]	0.001
6-h	285.1 [147.3–447.8]	244.0 [122.25–376.25]	345.5 [168.0–500.0]	0.007
24-h	148.1 [74.3–189.8]	107.0 [72.25–188.0]	139.5 [81.0–194.25]	0.324
72-h	38.7 [24–41]	30.0 [21.75–38.0]	32.0 [25.0–45.0]	0.040
Troponin I (TnI)				
Pre-PCI	1.4 [0.1–1.5]	0.04 [0.01–0.2]	1.29 [0.37–3.46]	0.001
6-h	17.7 [6.6–30.0]	11.43 [5.26–29.2]	25.0 [10.1–30.0]	0.001
24-h	12.9 [4.3–16.1]	7.65 [3.3–14.89]	10.42 [5.5–16.9]	0.575
72-h	3.4 [1.1–4.3]	2.21 [1.04–3.64]	3.1 [1.2–4.77]	0.074
In-hospital stay length (days)	6.2 [5–7]	6.0 [5.0–7.0]	5.0 [5.0–7.0]	0.265
Intrahospital complications	0 (0%)	0	0	1.000
Without	176 (88%)	88	88	1.000
Ventricular thrombus	4 (2%)	2	2	1.000
Pericardial effusion	5 (3%)	1	4	0.365
Ventricular fibrillation	11 (6%)	8	3	0.215
Pulseless electrical activity	1 (1%)	1	0	1.000
Cardiac tamponade	1 (1%)	0	1	1.000
Stroke	1 (1%)	0	1	1.000
In-hospital death	1 (1%)	0	1	1.000
Echocardiographic assessment				
In-hospital FSLV	35.0 ± 7.3	34.7 [29.55–38.42]	35.95 [32.6–40.15]	0.254
In-hospital EFLV	48.9 ± 7.4	50.0 [46.0–55.0]	48.0 [42.0–55.0]	0.024
In-hospital LVIDs	3.2± 0.6	3.2 [2.9–3.5]	3.15 [2.8–3.5]	0.647
In-hospital LVIDd	4.9 ± 0.4	4.9 [4.6–5.2]	4.9 [4.6–5.22]	0.823
In-hospital LVEDV	102.0 [81–117]	94.0 [84.0–115.25]	97.0 [78.5–121.5]	0.787
In-hospital LVESV	52.6 [39–63]	48.0 [40.88–60.0]	50.0 [36.25–65.0]	0.727
In-hospital MADd	3.0 [2.9–3.2]	3.0 [2.9–3.2]	3.0 [2.9–3.1]	0.374
In-hospital mitral regurgitation				
0	59 (30%)	26	33	0.352
0/1	5 (3%)	4	1	0.365
1	64 (32%)	38	26	0.095
1/2	31 (16%)	11	20	0.118
2	34 (17%)	19	15	0.572
2/3	7 (4%)	2	5	0.442

NYHA, New York Heart Association; CCS, Canadian Cardiology Society; PCI, Percutaneous Coronary Intervention; FSLV, Fractional Shortening of the Left Ventricle; EFLV, Left Ventricle Ejection Fraction; LVIDs, Left Ventricular Internal Dimension in Systole; LVIDd, Left Ventricular Internal Dimension in Diastole; LVEDV, Left Ventricular End-Diastolic Volume; LVESV, Left Ventricular End-Systolic Volume; MADd, Mitral Annulus Diameter Dimension.

**Table 2:** ECG characteristics at baseline and during the follow-up.

Variables	Whole Sample (*n* = 200) *n* (%) Mean ± SD Median [IQR]	Symptoms Duration of Less than 6 h (*n* = 100) Median [IQR] %	Symptoms Duration of Less than 6 h (*n* = 100 Median [IQR] %	*p* Value
Dynamics of the QRS complex (msec)				
Pre-PCI QRS width	100.5± 12.7	100.0 [90.0–110.0]	100.0 [93.5–110.0]	0.463
Post-PCI QRS width	98.8 ± 15.1	95.0 [85.0–100.0]	100.0 [90.0–110.0]	0.111
60-min QRS width	94.7 ± 13.3	86.5 [80.0–100.0]	100.0 [87.25–100.0]	0.008
72-h QRS width	89.8 ± 11.5	81.0 [80.0–90.5]	95.0 [85.0–100.0]	0.001
1-month QRS width	87.9 ± 10.6	80.0 [80.0–90.0]	90.0 [85.0–100.0]	0.001
6-months QRS width	87.7 ± 11.1	80.0 [80.0–90.0]	90.0 [80.0–100.0]	0.001
ST-segment elevation (mm)				
Pre-PCI	3.8[2–5]	3.5 [2.5–5.0]	3.0 [2.0–5.0]	0.064
Post-PCI	1.9 [0.5–3]	1.0 [0.0–2.5]	2.0 [0.5–3.0]	0.051
60-min	1 [0–2]	0.5 [0.0–1.0]	1.0 [0.0–2.0]	0.005
72-h	0.5 [0–0.5]	0.0 [0.0–0.5]	0.5 [0.0–1.0]	0.001
1-month	0.2 [0–0.1]	0.0 [0.0–0.0]	0.0 [0.0–0.5]	0.003
6-months	0.1 [0–0.1]	0.0 [0.0–0.0]	0.0 [0.0–0.0]	0.013
ST-segment resolution (>50%)				
Post-PCI	121 (61%)	72	49	0.001
60-min	98 (49%)	48	50	0.888
72-h	82 (41%)	39	43	0.666
1-month	52 (26%)	23	29	0.420
6-months	16 (8%)	7	9	0.794
Newly formed branch block				
No	175 (88%)	87	88	1.000
Transitory RBBB	2 (1%)	1	1	1.000
Transitory LBBB	9 (5%)	7	2	0.172
Transitory LAHB	4 (2%)	2	2	1.000
Permanent LAHB	5 (3%)	1	4	0.365
Permanent ILBBB	2 (1%)	0	2	0.477
Permanent RBBB	2 (1%)	2	0	0.477

PCI, Percutaneous coronary intervention; RBBB, Right Bundle Branch Block; LBBB, Left Bundle Branch Block; LAHB, Left Anterior Hemiblock; ILBBB, Incomplete Bundle Branch Block.

**Table 3:** MACE in STEMI patients.

Variables	Whole Sample (*n* = 200) *n* (%) Mean ± SD Median [IQR]	Symptoms Duration of Less than 6 h (*n* = 100) (%) Median [IQR]	Symptoms Duration of Less than 6 h (*n* = 100) (%) Median [IQR]	*p* Value
**6-months follow-up data**				
In-hospital death	1 (1%)	0	1	1.000
Out-of-hospital death	5 (3%)	1	4	0.365
Coronary event-related death	6 (3%)	1	5	0.214
Reinfarction of the treated vessel	1 (1%)	1	0	1.000
Manifested HF	28 (14%)	6	22	0.002
Stent thrombosis	4 (2%)	2	2	1.000
Clinically manifested restenosis	2 (1%)	0	2	0.477
Ventricular thrombus	1 (1%)	1	0	1.000
MACE	32 (16%)	9	23	0.012
Dual Antiplatelet Treatment				
Acetylsalicylic acid + Ticagrelor	79 (40%)	27	52	0.001
Acetylsalicylic acid + Clopidogrel	109 (55%)	69	40	0.001
Acetylsalicylic acid + Ticagrelor + Oral anticoagulant	2 (1%)	2	0	0.477
Acetylsalicylic acid + Clopidogrel + Oral anticoagulant	9 (5%)	2	7	0.172
Acetylsalicylic acid + Clopidogrel + Direct oral anticoagulant	1 (1%)	0	1	1.000
6-months NYHA				
1	171 (86%)	94	77	0.001
1/2	1 (1%)	1	0	1.000
2	19 (10%)	4	15	0.016
2/3	1 (1%)	0	1	1.000
3	2 (1%)	0	2	0.477
3/4	1 (1%)	0	1	1.000
6-months CCS				
0	174 (87%)	86	88	0.833
1	13 (7%)	8	5	0.566
2	6 (3%)	4	2	0.678
3	2 (1%)	1	1	1.000
Echocardiographic assessment				
6-months FSLV	35.7 [32.7–40.0]	35.8 [33.3–40.0]	36.0 [32.65–39.25]	0.572
6-months EFLV	51.8 [48.8–57]	55.0 [50.0–57.5]	50.0 [45.0–55.0]	0.001
6-months LVIDs	3.2 [2.9–3.5]	3.1 [2.8–3.45]	3.2 [2.9–3.5]	0.368
6-months LVIDd	5.0 ± 0.5	5.0 [4.65–5.3]	5.0 [4.5–5.35]	0.502
6-months LVEDV	104.0 ± 30	95.0 [80.0–115.0]	101.0 [80.5–124.0]	0.251
6-months LVESV	52.3 [37.5–60]	47.0 [37.0–52.5]	50.0 [40.0–67.0]	0.023
6-months MADd	3.1 [3–3.2]	3.1 [3.0–3.2]	3.0 [2.9–3.2]	0.183
6-months mitral regurgitation				
0	54 (27%)	28	26	0.873
0/1	7 (4%)	7	0	0.021
1	66 (33%)	36	30	0.452
1/2	29 (15%)	9	20	0.045
2	25 (13%)	13	12	1.000
2/3	10 (5%)	5	5	1.000
3	1 (1%)	0	1	1.000
3/4	2 (1%)	1	1	1.000
**6-years follow-up data**				
All-cause mortality	22 (11%)	7	15	0.114
Cardiovascular death	14 (7%)	6	8	0.782
Non-cardiovascular death	8 (4%)	1	7	0.071
Hospitalization due to HF	5 (3%)	3	2	1.000
Reinfarction	7 (4%)	6	1	0.124
Stroke	4 (2%)	3	1	0.614
Stent restenosis	4 (2%)	2	2	1.000
Re-PCI of the non-culprit vessel	10 (5%)	5	5	1.000
Number of days until the first MACE	1745	2287.0 [1874.0–2476.0]	1854.0 [615.0–2208.5]	0.001
Number of days until death	2063	2361.0 [2272.5–2484.0]	1942.0 [1601.75–2260.5]	0.001

HF, Heart Failure; MACE, Major Adverse Cardiovascular Events; NYHA, New York Heart Association; CCS, Canadian Cardiology Society; FSLV, Fractional Shortening of the Left Ventricle; EFLV, Left Ventricle Ejection Fraction; LVIDs, Left Ventricular Internal Dimension in Systole; LVIDd, Left Ventricular Internal Dimension in Diastole; LVEDV, Left Ventricular End-Diastolic Volume; LVESV, Left Ventricular End-Systolic Volume; MADd, Mitral Annulus Diameter Dimension; PCI, Percutaneous Coronary Intervention.

**Table 4:** Association of QRS complex width and MACE.

QRS Complex	MACE	Width [msec]	*p* Value	OR [95% CI]	*p* Value
Pre-PCI	No	100 [IQR 90–105]	0.026	1.028 [1.003–1.053]	0.029
	Yes	102.5 [IQR 90–110]			
Right after the procedure	No	95 [IQR 85–100]	0.010	1.026 [1.004–1.048]	0.020
	Yes	100 [IQR 90–110]			
1-h after the procedure	No	94 [IQR 85–100]	0.013	1.027 [1.003–1.052]	0.025
	Yes	95 [IQR 85–105]			
72 h after the procedure	No	85 [IQR 80–95]	0.020	1.031 [1.002–1.060]	0.034
	Yes	89 [IQR 80–100]			
1 month	No	85 [IQR 80–90]	0.034	1.042 [1.012–1.074]	0.007
	Yes	85 [IQR 80–100]			
6 months	No	85 [IQR 80–90]	0.041	1.038 [1.009–1.069]	0.011
	Yes	87.5 [IQR 80–100]			

MACE, Major Adverse Cardiovascular Events; PCI, Percutaneous coronary intervention.

**Table 5:** Association of ST-segment elevation and MACE in STEMI patients.

ST-Segment	MACE	Elevation [mm]	*p* Value	OR [95% CI]	*p* Value
Pre-PCI	No	3 [IQR 2–5]	0.018	1.61 [1.024–1.317]	0.020
	Yes	3 [IQR 2.5–5]			
Right after the procedure	No	1 [IQR 0–2.5]	0.001	1.296 [1.103–1.523]	0.002
	Yes	2 [IQR 0.75–3]			
1-h after the procedure	No	0 [IQR 0–1]	0.001	1.391 [1.111–1.741]	0.004
	Yes	0.5 [IQR 0.5–2]			
72 h after the procedure	No	0 [IQR 0–5]	0.004	1.500 [1.037–2.170]	0.031
	Yes	0 [IQR 0–0.75]			
1 month	No	0 [IQR 0–0]	0.001	3.256 [1.572–6.747]	0.001
	Yes	0 [IQR 0–0.5]			
6 months	No	0 [IQR 0–0]	0.003	2.972 [1.268–6.965]	0.012
	Yes	0 [IQR 0–0.25]			

MACE, Major Adverse Cardiovascular Events; PCI, Percutaneous coronary intervention.

**Table 6:** Multivariate model for QRS width pre-PCI and CPK and Troponin.

MACE	Odds Ratio	Std. Err.	z	P > z	[95% Conf.	Interval]
QRS pre-PCI	1.027325	0.0129865	2.13	0.033	1.002185	1.053096
CPK MB pre-PCI	1.003071	0.0034383	0.89	0.371	0.9963542	1.009832
TNI 0	1.012977	0.0970325	0.13	0.893	0.8395824	1.222181
_cons	0.0243191	0.0316703	−2.85	0.004	0.0018943	0.3122134

**Table 7:** Multivariate model for ST-segment elevation pre-PCI and CPK and Troponin.

MACE	Odds Ratio	Std. Err.	z	P > z	[95% Conf.	Interval]
ST-segment elevation pre-PCI	1.165351	0.0758857	2.35	0.019	1.025718	1.323993
CPK MB pre-PCI	1.002997	0.0034725	0.86	0.387	0.996214	1.009826
TNI 0	1.0225	0.0989468	0.23	0.818	0.8458492	1.236043
_cons	0.0243191	0.0316703	−2.85	0.004	0.0018943	0.3122134

## Data Availability

The data presented in this study are available on request from the corresponding author due to privacy reasons.

## Supplementary Materials

The following supporting information can be downloaded at: https://www.mdpi.com/article/10.3390/diagnostics14101042/s1, Table S1: Culprit vessel and treatment data; Table S2: Additional 6-month follow-up data.
