# Supplementary material for: The Role of QRS Complex and ST-Segment in Major Adverse Cardiovascular Events Prediction in Patients with ST Elevated Myocardial Infarction: A 6-Year Follow-Up Study
Source: Diagnostics (Basel). 2024 May 17;14(10):1042. doi: 10.3390/diagnostics14101042 (PMC11120035; doi:10.3390/diagnostics14101042)
Supplement: Supplementary file 1 [file diagnostics-14-01042-s001.zip › diagnostics-2917037-supplementary.pdf]

Table S1. Culprit vessel and treatment data

Variables	Whole sample (N = 200)	Symptoms duration of less than 6 hours (N = 100)	Symptoms duration of more than 6 hours (N = 100)	P value
**MI localization**				
**Wide-anterior**	26 (13%)	8	18	0,058
**Anteroseptal**	26 (13%)	9	17	0,141
**Anterolateral**	15 (8%)	7	8	1,000
**Anteroseptoapical**	10 (5%)	4	6	0,746
**Lateral**	4 (2%)	1	3	0,614
**Inferior**	60 (30%)	31	29	0,877
**Posterior-Inferior**	39 (20%)	29	10	**0,001**
**Posterior-inferior-lateral**	18 (9%)	9	9	1,000
**Circular**	2 (1%)	2	0	0,477
**PCI/CABG arteries**				
**LAD**	57 (29%)	21	36	**0,028**
**LAD+RCA**	22 (11%)	13	9	0,498
**LAD+RCX**	6 (3%)	4	2	0,678
**LAD+OM1**	1 (1%)	0	1	1,000
**LAD+OM2**	1 (1%)	0	1	1,000
**LAD+D1**	1 (1%)	1	0	1,000
**LAD+RIM**	1 (1%)	1	0	1,000
**RCA**	69 (35%)	43	26	**0,017**
**D1**	2 (1%)	1	1	1,000
**D1+RCA**	1 (1%)	0	1	1,000
**RCX+RCA**	6 (3%)	4	2	0,678
**RIA+RCX+RCA**	12 (6%)	7	5	0,766
**OM1**	6 (3%)	1	5	0,214
**OM2**	4 (2%)	1	3	0,614
**RCX**	7 (4%)	3	4	1,000
**PD**	1 (1%)	0	1	1,000
**RPL**	1 (1%)	0	1	1,000
**OM2+RCA**	1 (1%)	0	1	1,000
**RCA+RPL**	1 (1%)	0	1	1,000
**Infarct-Related Artery (IRA)**				
**RCA**	99 (50%)	63	36	**0,000**
**LAD**	75 (38%)	28	47	**0,009**
**D1**	3 (2%)	1	2	1,000
**PD**	2 (1%)	0	2	0,477
**OM1**	6 (3%)	1	5	0,214
**RCX**	10 (5%)	6	4	0,746
**OM2**	4 (2%)	1	3	0,614
**RPL**	1 (1%)	0	1	1,000

**Arterial segment**				
**Proximal**	77 (39%)	36	41	0,561
**Medial**	97 (49%)	46	51	0,571
**Distal**	26 (13%)	18	8	0,058
**Degree of stenosis - IRA**	99,29	100.0 [100.0 - 100.0]	100.0 [100.0 - 100.0]	0,105
**Cardiac dominance**				
**Right coronary artery**	181 (91%)	88	93	0,335
**Circumflex artery**	12 (6%)	8	4	0,372
**Both**	7 (4%)	4	3	1,000
**Door-to-balloon time (min)**	47.2	42.0 [31.5 - 54.25]	40.5 [34.75 - 55.0]	0,672
**Predilatation**	153 (77%)	73	80	0,317
**Direct implantation**	31 (16%)	15	16	1,000
**Thromboaspiration**	33 (17%)	23	10	**0,022**
**Stented arteries**				
**LAD**	64 (32%)	24	40	**0,023**
**LAD+RCA**	15 (8%)	8	7	1,000
**LAD+RCX**	4 (2%)	2	2	1,000
**LAD+OM1**	1 (1%)	0	1	1,000
**LAD+RIM**	1 (1%)	1	0	1,000
**LAD+D1**	1 (1%)	1	0	1,000
**RCA**	79 (40%)	51	28	**0,001**
**RCX+RCA**	6 (3%)	4	2	0,678
**PD**	1 (1%)	0	1	1,000
**RIA+RCX+RCA**	3 (2%)	1	2	1,000
**RCX**	8 (4%)	4	4	1,000
**D1**	3 (2%)	1	2	1,000
**RCA+RPL**	3 (2%)	1	2	1,000
**OM1**	5 (3%)	1	4	0,365
**OM2**	4 (2%)	1	3	0,614
**OM1+RCA**	1 (1%)	0	1	1,000
**OM2+RCA**	1 (1%)	0	1	1,000
**Compromised collateral vessel**	24 (12%)	15	9	0,277
**Kissing technique**				
**No**	184 (92%)	92	92	1,000
**PTCA-PTCA**	15 (8%)	7	8	1,000
**ST-ST**	1 (1%)	1	0	1,000
**Bifurcation technique**	1 (1%)	1	0	1,000
**Additional work-up**				
**No**	15 (8%)	72	81	0,182
**PCI during the same hospitalization**	184 (92%)	14	11	0,669
**PCI during the other hospitalization**	1 (1%)	11	7	0,459

**CABG during the other hospitalization**	1 (1%)	3	1	0,614
**Number of implanted stents**	1,4	1.0 [1.0 - 2.0]	1.0 [1.0 - 2.0]	0,105
**Total stent length (mm)**	30,1	25.5 [18.0 - 35.25]	26.0 [20.0 - 40.25]	0,067
**Information about the stented artery:**				
**Stent length (mm)**	22,6	21.0 [18.0 - 26.0]	23.0 [18.0 - 28.0]	0,055
**Stent diameter**	3,1	3.0 [3.0 - 3.5]	3.0 [3.0 - 3.5]	0,559
**Atmosphere**	13,3	12.0 [12.0 - 16.0]	14.0 [12.0 - 15.0]	0,604
**Type of the stent**				**0,010**
**Bare-metal stent**	111 (56%)	65	46	
**Drug-eluted stent**	89 (45%)	35	54	
**Stented coronary artery**				
**RCA**	97 (49%)	63	34	**0,000**
**LAD**	75 (38%)	28	47	**0,009**
**D1**	3 (2%)	1	2	1,000
**PD**	2 (1%)	0	2	0,477
**OM1**	6 (3%)	1	5	0,214
**RCX**	10 (5%)	6	4	0,746
**OM2**	4 (2%)	1	3	0,614
**RIM**	2 (1%)	0	2	0,477
**RPL**	1 (1%)	0	1	1,000
**Patients with optimal outcome**	200 (100%)	100	100	1,000
**Complications**				
**Without**	193 (97%)	98	95	0,442
**Coronary artery dissection**	6 (3%)	2	4	0,678
**Death in a catheterization laboratory**	1 (1%)	0	1	1,000
**Pre-PCI TIMI flow**				
**0**	166 (83%)	84	82	0,851
**0/1**	3 (2%)	1	2	1,000
**1**	6 (3%)	4	2	0,678
**1/2**	2 (1%)	1	1	1,000
**2**	13 (7%)	6	7	1,000
**2/3**	4 (2%)	2	2	1,000
**3**	6 (3%)	2	4	0,678
**Post-PCI TIMI flow**				
**0**	1 (1%)	1	0	1,000
**1**	1 (1%)	1	0	1,000
**1/2**	3 (2%)	0	3	0,245
**2**	46 (23%)	19	27	0,240
**2/3**	10 (5%)	5	5	1,000

**3**	139 (70%)	74	65	0,219
**TIMI MPG**				
**4**	127 (64%)	64	63	1,000
**3**	59 (30%)	28	31	0,756
**2**	9 (5%)	6	3	0,495
**1**	5 (3%)	2	3	1,000
**GP IIB/IIIa**	75 (38%)	40	35	0,559
**Fibrinolysis due to no reflow**	1 (1%)	1	0	1,000

PCI, Percutaneous Coronary Intervention; CABG, Coronary Artery Bypass Grafting; LAD, Left Anterior Descending artery; RCA, Right Coronary Artery; RCX, Circumflex Artery; OM1, First Obtuse Marginal artery; OM2, Second Obtuse Marginal artery; D1, First Diagonal artery; RIM, Ramus Intermedius artery; RIA, Right Interventricular Artery; PD, postero descending; RPL – ramus postero lateralis; PTCA, Percutaneous Transluminal Coronary Angioplasty; TIMI, Thrombolysis In Myocardial Infarction; MPG, Myocardial Perfusion Grade; GP, glycoprotein;

Table S2. Additional 6-month follow-up data

Variables	Whole sample (N = 200)	Symptoms duration of less than 6 hours (N = 100)	Symptoms duration of more than 6 hours (N = 100)	P value
**Ischemia assessment**				
**Wasn't performed**	124 (62%)	53	71	**0,013**
**Positive ergometric test**	2 (1%)	0	2	0,477
**Negative ergometric test**	60 (30%)	41	19	**0,001**
**Positive stress echocardiography**	2 (1%)	1	1	1,000
**Negative stress exhocardiography**	10 (5%)	5	5	1,000
**Positive SPECT**	1 (1%)	0	1	1,000
**Negative SPECT**	1 (1%)	0	1	1,000
**6-months reintervention due to possible ischemia**	7 (4%)	3	4	1,000
**Atrial Fibrillation**				
**Without**	176 (88%)	88	88	1,000
**New onset (transitory) during hospitalization**	16 (8%)	9	7	0,794
**New onset (permanent) during hospitalization**	4 (2%)	1	3	0,614
**New onset (permanent) during the follow-up**	1 (1%)	1	0	1,000
**Permanent**	3 (2%)	1	2	1,000

SPECT, Single Photon Emission Computed Tomography
